# Quasi-Periodic Crystals—The Long Road from Discovery to Acceptance^*^

**DOI:** 10.5041/RMMJ.10102

**Published:** 2013-01-30

**Authors:** Daniel Shechtman

**Affiliations:** Nobel Prize Laureate in Chemistry, 2011. Philip Tobias Professor of Materials Science, Department of Materials Science and Engineering, Technion–Israel Institute of Technology, Haifa, Israel

**Keywords:** Quasi-periodic crystals, crystallography, periodicity, paradigm shift

Difficult things take a long time, impossible things—a little longer.

(∼André A. Jackson)

For all things difficult to acquire, the intelligent man works with perseverance.

(Lao Tzu)

## INTRODUCTION

Three surprising discoveries on the nature of matter and its properties were published in the mid-1980s. All these discoveries led to Nobel prizes. The first discovery was in 1985, when fullerenes were discovered by Robert F. Curl Jr, Sir Harold W. Kroto, and Richard E. Smalley. Fullerenes, also known as *buckyballs*, are spherical molecules composed of carbon atoms. The discovery of fullerenes launched the field of nano-materials, one of the fastest-growing fields in chemistry today. In 1996, 11 years after the publication of the discovery, the three researchers were jointly awarded the Nobel Prize in chemistry. No controversy surrounded this discovery.

In 1986, two IBM researchers, Karl Müller and Johannes Bednorz, discovered high-temperature superconductive materials. Although superconductivity was first discovered in 1911, nobody expected to see this phenomenon at the relatively high temperatures of liquid nitrogen. In 1987, one year after publishing their discovery, the two researchers were awarded the Nobel Prize in physics. Again, no controversy surrounded this discovery, and, as the short period of time between the discovery and awarding of the prize shows, the discovery was enthusiastically embraced by the scientific community.

Publication of the third discovery pre-dates the publication of the other two discoveries. I published the discovery of quasi-periodic crystals in 1984 and was awarded a Nobel Prize in 2011, 27 years after the discovery. Unlike the previous two discoveries, this discovery was met with fierce opposition and a substantial amount of controversy.

What was so controversial about this discovery that it raised the antagonism of so many people in the scientific community? Why would Linus Pauling, a twice-awarded Nobel Laureate and one of the greatest chemists of the twentieth century, state: “There is no such thing as quasi-crystals, only quasi-scientists”? In order to answer these questions, I must first give a short introduction to crystallography. For that purpose, I will define three basic terms in crystallography: order, periodicity, and rotational symmetry.

## UNDERSTANDING CRYSTALLOGRAPHY

### Order

**Figure 1:**
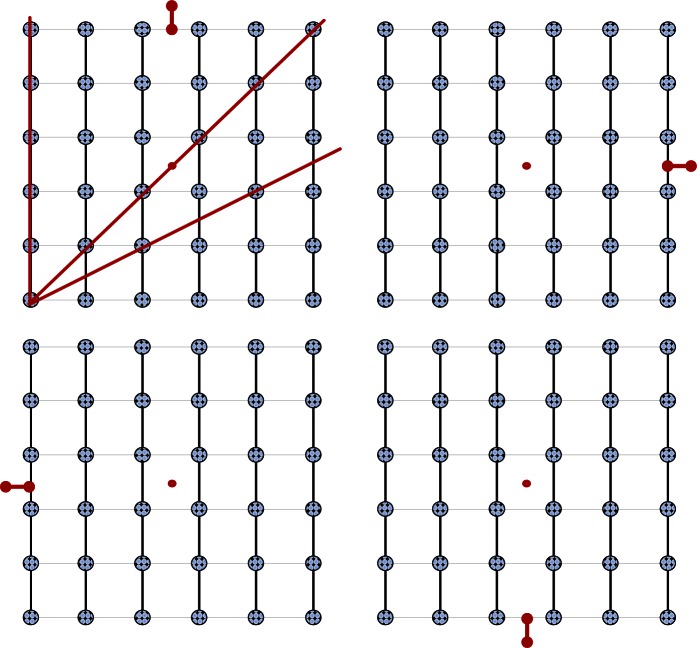
**Example of rotation, order, and symmetry in an atomic lattice.**

Crystals are solids that have an atomic structure of an indefinitely extended, three-dimensional order. A simple two-dimensional ordered lattice is shown in Figure 1. The continued order of this lattice is evident in all directions.

### Periodicity

The periodicity of the lattice is defined by the lengths and mutual orientations of the three lattice vectors that enclose the pattern. As can be seen in Figure 1 (top left), periodicity exists when the distance between any two adjacent points on a straight vector is the same.

### Rotational Symmetry

**Figure 2:**
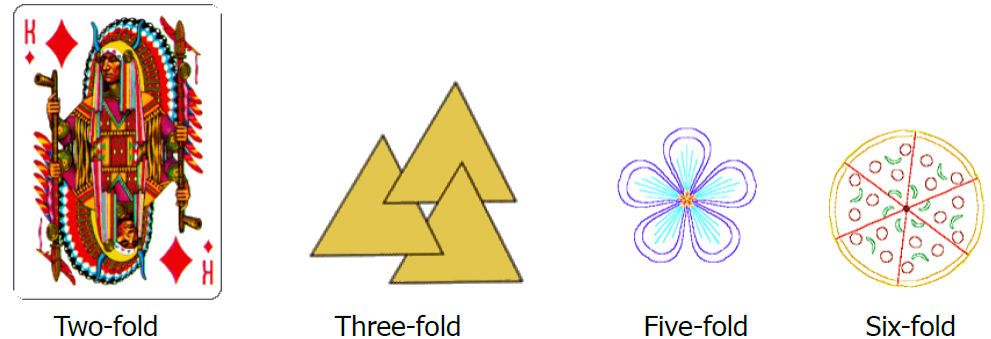
**Objects with a two-, three-, five-, and six-fold rotational symmetry.**

An object that has rotational symmetry is an object that looks identical after it is rotated. The lattice in Figure 1 is identical if we rotate it by 90°, 180°, 270°, or 360°. Therefore, this lattice has a four-fold rotational symmetry. Figure 2 shows objects that have two-, three-, five-, and six-fold rotational symmetry.

## THE HISTORY OF MODERN CRYSTALLOGRAPHY

The modern science of crystallography actually started in 1912, with an experiment by Max von Laue, a German scientist, who proved two amazing things in one experiment. First: that X-rays are electromagnetic waves with a wavelength of about 10^−10^ m, and second: that the internal structure of crystals is regular, and that it is arranged in three-dimensional structures. William Lawrence Bragg and his father, Sir William Henry Bragg, developed an equation, aptly named Bragg’s law, which measures the angles and spacing between the atoms of the crystal, thus allowing the crystalline structure to be constructed from the scattered dots seen on an X-ray diffraction pattern.

Zinc sulphate was the first crystal studied by von Laue. This crystal was not only ordered but was periodic as well. Von Laue analyzed many other crystals and found that they all shared these two properties. For 70 years, from 1912 to 1982, hundreds of thousands of crystals were studied, all of which were ordered and periodic. There were no exceptions. Due to this overwhelming empirical evidence, a paradigm was developed for the definition of a crystal. For example, a well-known textbook by B. D. Culity, *Elements of X-Ray Diffraction* (1959), defines a crystal as “a solid composed of atoms arranged in a periodic pattern in three dimensions.” This definition was not developed from a theoretical model but came from repeated observations.

**Figure 3:**
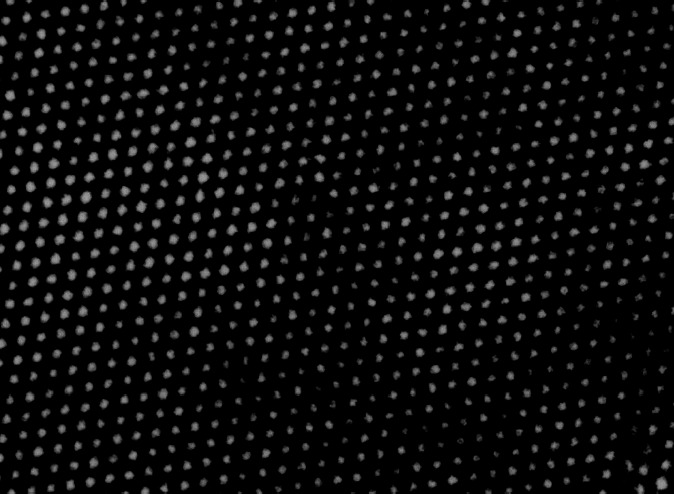
**Atoms in a diamond as seen under an electron microscope.**

Charles Kittel writes in his influential textbook *Introduction to Solid State Physics* the following: “We can make a crystal from molecules which individually have a five-fold rotation axis, but we should not expect the lattice to have a five-fold rotation axis.” Crystals do not have to be made of atoms with repeating periodic patterns. Crystals can be made up of molecules, even very large molecules such as proteins, with repeating periodic patterns. The individual molecules of this crystal can have a five-fold rotational symmetry, but the crystal as a whole cannot have a five-fold rotational symmetry. As an illustration, a connected array of pentagons cannot fill the entire plane without leaving gaps. Therefore, it was assumed that there are no crystals with a five-fold rotation axis and no crystals with more than a six-fold rotational axis. For example, a diamond (Figure 3) is an ordered and periodic crystal. The rotational symmetries that are allowed in this crystal are one, two, three, four, and six. It has no five-fold rotational axis and no rotational axis above six.

## THE DISCOVERY OF QUASI-PERIODIC CRYSTALS

Science advances through discoveries. Most discoveries are incremental in nature, and although they broaden our horizons and are beneficial to mankind, they do not break any norms nor do they cause paradigm shifts. Occasionally, an interesting discovery comes along and causes a shake-up in the scientific community. Although these discoveries often end up being artifacts, sometimes they are genuine discoveries, causing scientists to rethink the tenets of their field and change their paradigms.

**Figure 4:**
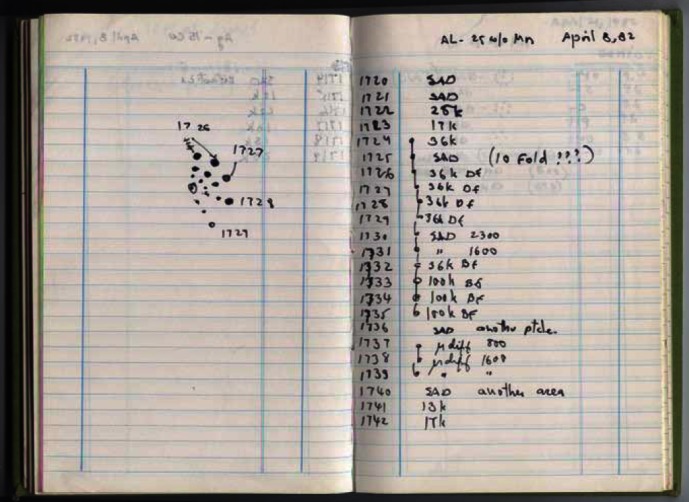
**Logbook of Professor D. Shechtman.**

**Figure 5:**
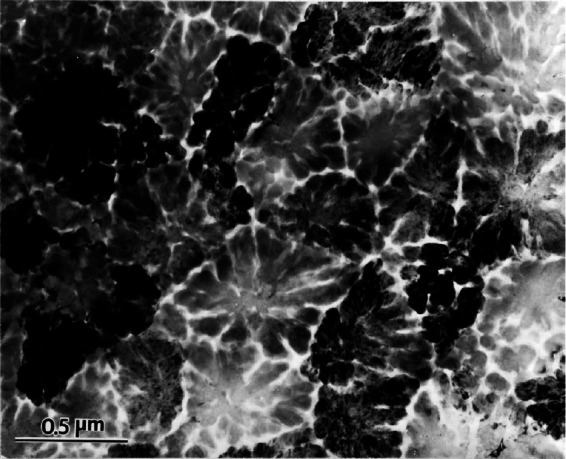
**First view of the icosahedral phase. Heavy diffraction is noted by the black crystals.**

One such discovery occurred to me on April 8, 1992, at 10.00 a.m. (Figure 4). I was looking at an electron diffraction pattern of an aluminum manganese compound that formed in a rapidly solidified alloy with composition close to Al_6_Mn, taken by an electron microscope. Electron diffractions contain the same information as X-ray diffractions. While looking at this pattern, I noticed two very strange things: first, this compound had a 10-fold rotational axis and, second, it had no periodicity (Figure 5). If the distance between two spots is taken as the periodic distance and is multiplied by two, we should expect to reach the next diffraction spot. However, in this diffraction pattern (Figure 5), we reached nothing. Therefore, this crystal had no periodicity. This crystal violated both laws of crystallography of the time: it had no periodicity, and it had a 10-fold rotational symmetry.

However, this crystal did have quasi-periodicity. The ratio of the distances from the central spot to two spots that are adjacent to each other equals the Fibonacci number τ, which is also known as the golden mean or golden ratio. This number is an irrational number of approximately 1.618. It is also the ratio of sequential elements of the Fibonacci sequence (0, 1, 1, 2, 3, 5, 8, 13, 21 …) which approaches the golden ratio asymptotically. The common denominator between the Fibonacci series and quasi-periodical crystals is that there is no motif of any size which repeats itself. However, they both have governing rules enabling them to continue indefinitely. The Fibonacci series is an example of quasi-periodicity in one dimension.

**Figure 6:**
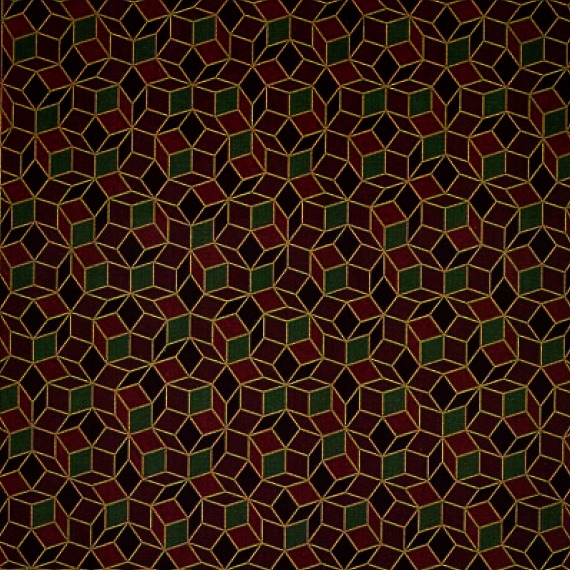
**Penrose tiles.**

A two-dimensional example of quasi-periodicity is Penrose tiles, named after Professor Roger Penrose (Figure 6). If the colors are ignored, only two types of tiles remain: a thin rhombus and a thick rhombus. A plane can be tiled according to specific rules, and the result is a quasi-periodic array. Roger Penrose proposed that this set of two tiles could only produce non-periodic tiling. Alan L. Mackay showed that the diffraction pattern of the Penrose tiling has a five-fold symmetric pattern.

**Figure 7:**
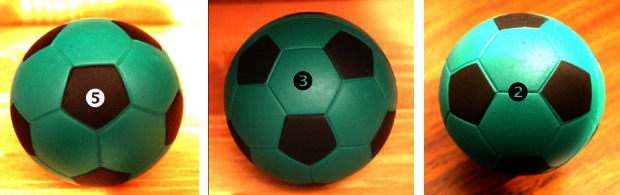
**The icosahedron’s main rotational symmetries.**

My discovery of quasi-periodical crystals occurred while I was staying at the National Bureau of Standards, where I was on sabbatical from 1981 to 1983. The reaction to my discovery was mixed. One colleague literally came to my office with a textbook proving that this pattern was just not possible. My group leader at the time called me a disgrace and asked me to leave his group. On the other hand, Professor John Cahn, an eminent researcher in thermodynamics, came to me and said, “Danny, this material is telling us something and I am challenging you to find out what it is.” When I returned to the Technion in 1984, I collaborated with Professor Ilan Blech on the first quasi-periodical crystal paper. Professor Blech developed a model that described how such material could form. The model is derived from pentagonal symmetry, which is one of the rotational symmetries of three-dimensional bodies called icosahedrons. To simplify, imagine a football, or as the Americans call it, a soccer ball. This ball is made of pentagonal and hexagonal patches (Figure 7) and clearly has five-fold, three-fold, and two-fold symmetries (Figure 7; left, middle, and right panels, respectively). Icosahedral symmetry also has these features. This was the model that we proposed for our crystal.

In mid-1984, we sent the paper to the *Journal of Applied Physics*, and within two weeks it was returned with a letter stating that the Journal was not interested in this manuscript and that the topic would not interest the community of physicists. They suggested sending the manuscript to a metallurgical journal. I subsequently sent this paper to the journal *Metallurgical Transactions*, where it was accepted for publication. However, the paper was scheduled to be published only in June of 1985. In the summer of 1984 I was back at the National Bureau of Standards. Professor Cahn suggested writing a shortened version of the same paper and submitting it to a journal that would publish it more quickly. We wrote a shortened version together with Dr. Denis Gratias, a mathematical crystallographer from France; the paper was subsequently published on November 12, 1984 in the journal *Physical Review Letters.*

Since I knew my discovery was controversial, I wanted anyone who had the appropriate equipment to be able to prepare this crystal and see the results under an electron microscope. I was therefore meticulous in providing all the details. A few days after publication, I began receiving phone calls from researchers around the world saying that they too had seen what I saw. I was witnessing a growing community of powerful, amazing, young, avant-garde, quasi-periodic scientists. Eminent physicists, chemists, mathematicians, and material scientists around the world started creating the science of quasi-periodic materials. However, changing paradigms is never easy, and this case was no different.

## THE PARADIGM SHIFTS SLOWLY

To date, most crystallographers use X-ray diffracttion as their primary and often exclusive research tool. They believe that X-ray diffraction is more precise than electron microscopy crystallography. I was in the minority, using electron microscopy to study crystals. The minimum size of crystals used in X-ray crystallography was a fraction of a millimeter, while crystals used in electron microscopy can be nanometers in size, and the size of my crystals was about one micron. The International Union of Crystallographers, a very strict and precise group of mathematical crystallographers, was not willing to entertain the notion of quasi-periodic crystals until we produced X-ray diffraction patterns.

Worse yet, Linus Pauling, one of the greatest chemists of the twentieth century and the only person to win two individual Nobel prizes, was adamantly against the notion that quasi-periodical crystals exist. In the October 10, 1985 issue of *Nature* he published a rebuttal article claiming that these irregularly shaped crystals were formed by a natural process called icosahedral twinning, several intergrown crystals whose shared boundary gives rise to a composite diffraction pattern. In paper after paper published in prestigious journals such as the *Proceedings of the National Academy of Sciences* and *Science News*, Pauling continued his crusade to debunk the entire notion of quasi-periodical crystals. During an American Chemical Society conference at Stanford, in front of thousands of scientists, Pauling proclaimed “Danny Shechtman is talking nonsense. There is no such thing as quasi-crystals, only quasi-scientists.”

**Figure 8:**
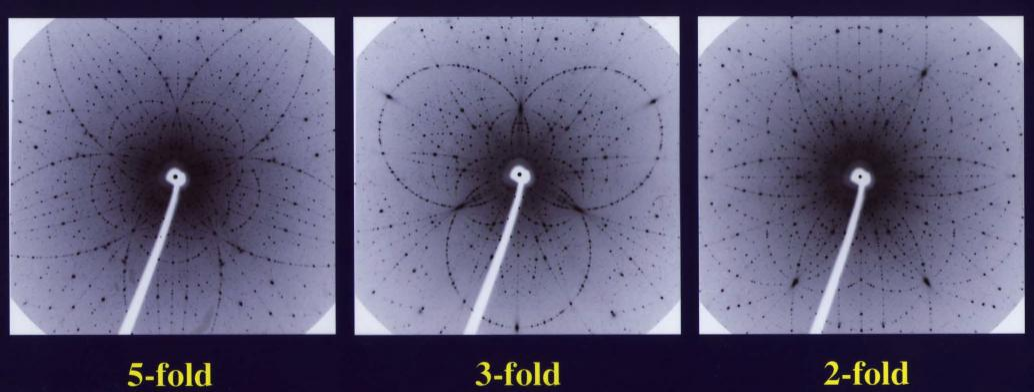
**X-ray transmission Laue photograph of the quasi-crystal i-ZnMgHo, courtesy of An-Pang Tsai, Japan.**

It took three years for scientists to develop methods to grow these crystals to the size needed for X-ray diffraction. I received X-ray diffraction patterns from scientists in Japan and France who confirmed that these crystals were quasi-periodical and that their structure had icosahedral symmetry (Figure 8). When I presented these findings at the International Union of Crystallographers meeting in Perth, Australia in 1987, they said, “OK, Danny, now you are talking.”

A committee was formed that redefined crystals. This represented a very significant paradigm shift. In 1992, the International Union of Crystallographers came out with a new definition of crystals which reads as follows: “… By crystal we mean any solid having an essentially discrete diffraction diagram, and by aperiodic crystal we mean any crystal in which three dimensional lattice periodicity can be considered to be absent.” This is a humble definition, an open definition. Humble scientists are good scientists. They are open to listen to new ideas. They ask questions and critique, but do not knock down and dismiss ideas just because they do not fit their preconceived notions.

Supported by the American Chemical Society members, Linus Pauling refused to accept the growing evidence for quasi-periodic crystals until his death in 1994. By the late 1980s, the tide was already turning regarding the existence of quasi-periodical crystals. All of Pauling’s alternative models which purported to explain the quasi-periodical crystals were proven wrong, and after Pauling’s death opposition to my discovery ceased. Not long after publication of my first article on the subject, I was given a book called *The Structure of Scientific Revolutions* written by the philosopher of science Thomas Kuhn. This book deals with the process by which scientific paradigms are produced and replaced. I warmly endorse this book because I have lived through several of the stages described in the book.

## WHY WEREN’T QUASI-PERIODIC MATERIALS DISCOVERED EARLIER?

**Figure 9:**
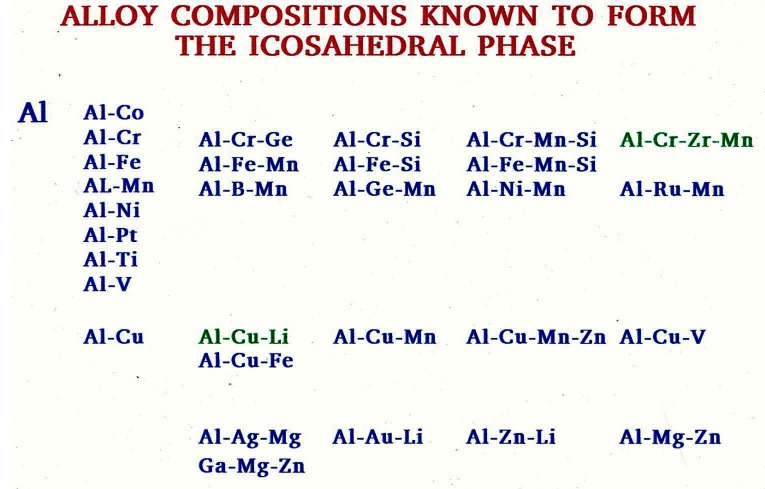
**Quasi-periodic materials with aluminum in them.**

Why were quasi-periodic materials not discovered before 1982? For 70 years, hundreds of thousands of crystals were discovered and analyzed by X-ray crystallographers, and not one saw quasi-periodic materials. Quasi-periodic materials are not rare. There are hundreds upon hundreds of them. A partial list of some of the quasi-periodical crystals based on aluminum can be seen in Figure 9. Other quasi-periodical crystals are based on copper, iron, nickel, and more. Clearly, these materials are abundant and not esoteric. It is true that many quasi-periodic materials transform to a periodic structure only at high temperatures, but many are thermodynamically stable at lower temperatures as well. These metallic alloys are also easy to produce by all the common methods used in industry such as casting, rapid solidification, single growth crystal, electrode position, chemical vapor deposition (CVD), and physical vapor deposition (PVD).

I would like to present my own subjective answer to this question. As previously mentioned, quasi-periodic crystals are small. Therefore, the only tool that could have discovered them is a transmission electron microscope (TEM). The vast majority of crystallographers work with X-ray diffractions; hence, the number of scientists who could have made this discovery is automatically limited. Like any other sophisticated tool, it is not enough to know how to use the TEM. To get these results, you must be an expert at using the TEM, and that narrows the number of potential discoverers even more. In addition, three important qualities are required of scientists who come across something unexpected: tenacity, courage, and belief. Professionals in any field, and so much more in science, should be their own worst critic. Once a discovery is made, one should go over the results, repeat the experiments, and make sure that the results are real and not artifacts. Once the results have been validated, the researcher should stand tall and defend them. This takes courage and tenacity, and often large quantities of both. However, the rewards for whoever walks this path are great.

I would like to share an anecdote about missed opportunities. During one of the conventions that I attended, I was approached by a European professor who told me that while he was going through the data of his students, he saw a slide with the diffraction pattern I had seen. To his amazement, the date on the slide predated my discovery. The professor contacted his former student who now holds a leading management position in the industry and asked him if he realized that he had seen the same diffraction pattern that I saw. The student answered in the affirmative. The professor then asked the student why he hadn’t shown his results at the time, and the student answered that if he had shown his results to the professor, he would have been asked to stay on for two more years to research that discovery, and he was not interested in doing so.

## LESSONS THAT I LEARNED

Finally, I would like to re-emphasize some of the lessons that life has taught me.

**Be a professional:** In any endeavor that you choose in any field, strive to be the best. Choose what you like or what you are good at, and become an expert in that field. I promise you, you will have a wonderful career.

**Tenacity:** If you discover something, hold on to it like a Rottweiler, and do not let go until you analyze what it is. In most cases, it will be an artifact, but in some cases, you will have made a great discovery. Do not let go.

**Believe in yourself:** If you have mastered your field, believe in yourself. Be your own worst critic, but if you have thoroughly checked your results and verified that they are real, take pride in your discovery and defend it.

**Courage:** Last but not least, you must have courage. Even when the top leaders in your field say that you are talking nonsense, you must have the courage to say that they are wrong.

## Abbreviations:

CVD: chemical vapor deposition;
PVD: physical vapor deposition;
TEM: transmission electron microscope
